# Empyema in a Woman with Cystic Fibrosis: A Cautionary Tale

**DOI:** 10.1155/2013/159508

**Published:** 2013-03-05

**Authors:** Anne Coates, Oren Schaefer, Karl Uy, Brian P. O'Sullivan

**Affiliations:** ^1^Pediatric Pulmonary Division, Lucile Packard Children's Hospital at Stanford, 770 Welch Road, Suite 350, Palo Alto, CA 94304, USA; ^2^Mass Lung and Allergy, PC, 33 Oak Avenue, Worcester, MA 01605, USA; ^3^Division of Thoracic Surgery, University of Massachusetts Medical School and UMass Memorial Health Care, 55 Lake Avenue, Worcester, MA 01655, USA; ^4^Department of Pediatrics, University of Massachusetts Medical School and UMass Memorial Health Care, 55 Lake Avenue, Worcester, MA 01655, USA

## Abstract

Cystic fibrosis (CF) is a disease which predisposes individuals to recurrent infective exacerbations of suppurative lung disease; however, empyema is a rare complication in these patients. Empyemas secondary to *Staphylococcus aureus* and *Burkholderia cepacia* have been described in patients with CF. We report the case of pleural empyema with mixed *S. aureus* and *Pseudomonas aeruginosa* infection in a 34-year-old woman with CF, which was managed with ultrasound-guided pigtail catheter insertion, fibrinolysis, and antibiotic therapy. Physicians should be aware of this unusual complication in CF patients, especially those receiving an immunosuppressive therapy.

## 1. Introduction

Cystic fibrosis (CF) is an autosomal recessively inherited genetic disorder caused by mutations of the CF transmembrane conductance regulator (CFTR) gene. Abnormalities of the respiratory epithelium result in dehydrated, thickened secretions which provide a favorable environment for infection [1]. Although at one point considered a pediatric disease, as of 2008 greater than 45% of United States CF patients were greater than 18 years of age [2]. Typically, airway infection starts with *H. influenzae* and *S. aureus* with progression to chronic infection with mucoid *Ps. aeruginosa* in the vast majority of patients. Infection with more than one organism is common.

Parapneumonic effusion is an accumulation of exudative pleural fluid associated with an ipsilateral pulmonary infection. This is a relatively common consequence of pneumonia in those without CF, occurring in 20–40% of non-CF patients admitted to the hospital with pneumonia [3]. An effusion is referred to as an empyema, when the concentration of leucocytes becomes macroscopically evident as thick and turbid fluid. A positive pleural fluid gram stain or culture also defines an empyema. CF patients rarely develop parapneumonic effusions or empyemas, despite their chronic airway infection. Taussig et al. [4] described 4 infants with CF who developed empyema secondary to *S. aureus* infection. Others have reported empyema in post-lung transplant patients [5, 6]. There has also been a single case report of pleural empyema in an immunocompetent adolescent with CF, chronically infected with *Ps. aeruginosa* and *S. aureus* [7].

We present a case of pleural empyema in an adult with CF who had mild lung disease at baseline and was not a transplant recipient. She was, however, receiving low-dose immunosuppression for treatment of allergic bronchopulmonary aspergillosis (ABPA). As anti-inflammatory therapy is further adopted as part of routine CF care, it is likely that this complication will become more common.

## 2. Case Report

CR is a 34-year-old female with CF homozygous for the F508del mutation whose sputum cultures had been positive for both *Ps. aeruginosa* and *S. aureus* for many years. Her CF was complicated by allergic bronchopulmonary aspergillosis (ABPA), marked seasonal allergies, and asthma. Her treatment regimen consisted of alternate day prednisone (20 mg), alternate week omalizumab (300 mg), itraconazole 100 mg bid, fluticasone 110 mcg/puff, 2 puffs bid, and azithromycin 500 mg orally every Monday-Wednesday-Friday. She chose not to use inhaled 7% hypertonic saline, rhDNase, or inhaled tobramycin. She used chest physiotherapy intermittently.

The patient was in her usual state of health (baseline FVC at 99% predicted and FEV_1_ at 85% predicted) until she developed intermittent, left-sided sharp chest pain at rest. She denied fever, hemoptysis, numbness, diaphoresis, paresthesias, left arm pain, or abdominal discomfort. The pain was worsened with cough. Upon admission to the hospital, a chest radiograph was consistent with a left pleural effusion and consolidation in the left mid and lower zones. There were also new inflammatory changes in the right mid zone.

**Figure 1:**
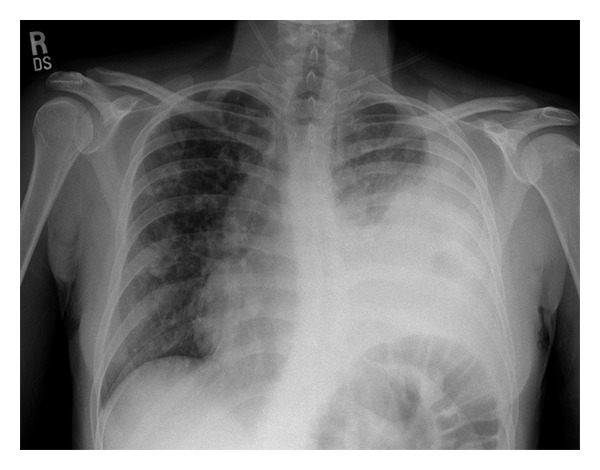
Chest radiograph showing left-sided pleural effusion shortly after admission.

**Figure 2:**
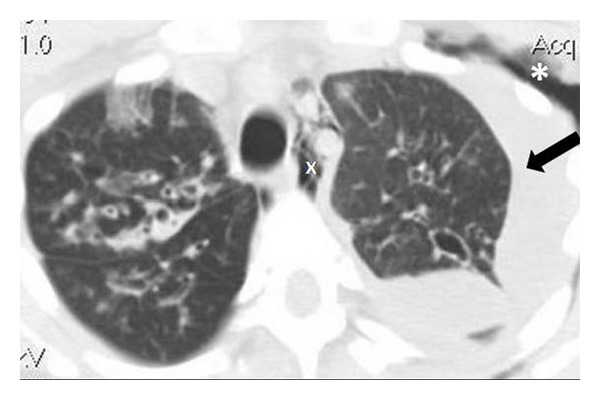
CT scan of the chest demonstrating pleural effusion (arrow), subcutaneous emphysema (asterisk), and pneumomediastinum (x). Subcutaneous air and mediastinal air are likely secondary to instrumentation.

The patient started on intravenous tobramycin and imipenem to treat the organisms known to be in her sputum. Despite appropriate therapy, on hospital day number 3, the patient developed increased respiratory distress and a temperature elevation to 39.4°C. A repeat chest X-ray film demonstrated a significant increase in the left pleural effusion and consolidation in the left lower lobe (Figure 1). A 6-French pigtail catheter was placed under ultrasound guidance on hospital day number 4, and pleural fluid was sent for microbiology culture. Pleural fluid analysis revealed a pleural fluid pH = 6.61, glucose < 5 mg/dL, LDH = 4531 IU/L, and total protein = 3.9 g/dL. Serology obtained at the time of the catheter placement revealed a total protein level =4.4 g/dL. There was no available serum LDH. A CT scan of the chest revealed a large partly loculated left pleural effusion with marked subcutaneous emphysema and pneumomediastinum (Figure 2). The pleural fluid culture was positive initially for *S. aureus*, and vancomycin was added to her antibiotic regimen. Subsequently, *Ps. aeruginosa* was also isolated from the pleural fluid. The patient underwent a second ultrasound-guided insertion of an 8-French pigtail catheter on hospital day number 5. Because of limited drainage, 10 milligrams of tissue plasminogen activator (tPA) mixed into 100 mL of normal saline were instilled into one of the chest tubes on both hospital days number 6 and number 7 with a resultant marked increase in fluid drainage. Her respiratory status improved significantly following this drainage. Serial chest-imaging studies performed throughout the hospitalization revealed improvement of the pleural fluid collection and subcutaneous emphysema. The pleural catheters were removed on hospital days 9 and 11, respectively, and she was ultimately discharged in stable condition in room air on day 15. She received an 18-day course of cefepime and 28-day course of inhaled tobramycin at home. 

**Figure 3:**
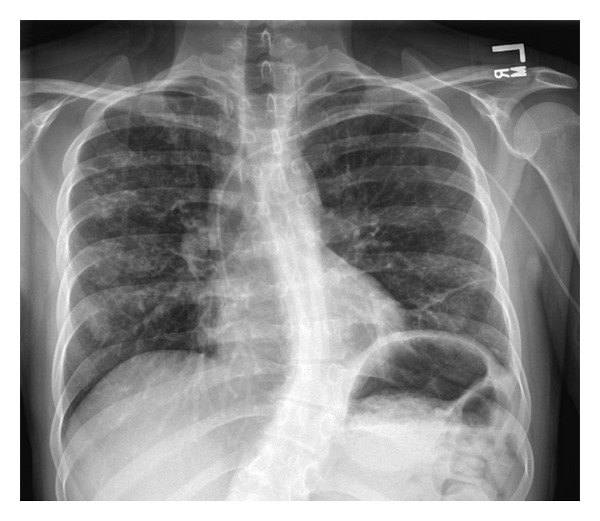
Chest radiograph taken two weeks after discharge from the hospital shows marked improvement of pleural and parenchymal disease. Central line for infusion of antibiotics is visible.

The patient was seen in pulmonary clinic 2 weeks after her hospital discharge where she was found to have an FVC of 90% predicted and FEV_1_ of 73% predicted, which were slightly below her baseline pulmonary function. A chest radiograph revealed near complete resolution of the left pleural effusion with minimal fluid remaining (Figure 3). Two months following her hospital discharge, the patient felt markedly improved, and her pulmonary function (FVC of 98% predicted and FEV_1_ of 79% predicted) was returning toward her baseline. 

## 3. Discussion

Empyema is by definition pus in the pleural space. The bacteriology of empyema is varied in non-CF versus CF patients and community- versus hospital-acquired infections. In a United Kingdom study of 300 non-CF patients with complicated parapneumonic effusion and empyema, the commonest causes of community-acquired pleural infections were the *Streptococcus milleri* group, *S. pneumonia,* and *Staphylococci*, sometimes with associated anaerobes. Cases of hospital-acquired infection in the same series were mostly caused by methicillin-resistant *S. aureus, S. aureus, Enterobacteria,* or *Enterococcus* [8]. 

Empyema is a rare but recognized complication in patients with CF. In these patients the causative organisms have been reported to be *S. aureus, P. aeruginosa*, and *Burkholderia cepacia*. Although first reported as a *S. aureus* infection in 4 infants between the ages of three weeks and eight months by Taussig et al. [4], there have been a few case reports of empyema in adolescents and adults. Empyema secondary to *Burkholderia gladioli* septicaemia and empyema necessitatis caused by *B. cepacia* have been reported in CF adults after lung transplantation [5, 6]. *Ps. aeruginosa* empyema in a 20-year-old with CF on long-term oral corticosteroid therapy was also reported [9]. Additionally, a 14-year-old patient with CF and hypersplenism developed an empyema [7].

Autopsy examination demonstrates the presence of pneumonia with parenchymal involvement (not just airway involvement) in the vast majority of CF patients [10]; yet extension into the pleural space is uncommon in this population. Reasons for this are unclear, but likely include host and pathogen interactions. *S. aureus* adheres predominantly to the mucus in CF airways rather than to airway epithelium [11], making it unlikely to migrate into the pleural space. Additionally, it is typical for CF patients to have elevated levels of specific antibodies to *Ps. aeruginosa, S. aureus,* and *S. pneumoniae* [12, 13] and a hyperimmune response to bacteria and bacterial endotoxins [14]. Although this enhanced inflammatory response likely contributes to lung destruction over time, it may serve as a protective mechanism in terms of extrapulmonary spread of bacteria. 

The common link among the few reported cases of empyema in patients with CF appears to be an alteration in this enhanced immune response. The suppressive effect of pre-and post-transplantation immunomodulator therapy and chronic corticosteroid use have been hypothesized as predisposing causes leading to empyema in several cases, whereas hypersplenism and the nadir in immunoglobulin-G levels during the transition from maternal to host-derived immunoglobulins may have been responsible for the other cases. In addition, selective antifungal therapies can suppress neutrophil response [15]. Our patient was on chronic alternate day prednisone at a relatively low dose (20 mg), itraconazole, and was receiving the anti-IgE antibody, omalizumab. It is unclear whether this degree of immunosuppression would be sufficient to predispose to a parapneumonic process. 

A growing number of anti-inflammatory agents are being introduced into the CF therapeutic armamentarium [16, 17]. As more patients are exposed to drugs that decrease the function of polymorphonucleocytes, inhibit proinflammatory cytokines, or decrease metabolites of arachidonic acid, it seems likely that they will be vulnerable to spread of infection beyond the lungs. Already, one study of an anti-inflammatory agent was halted early due to increased exacerbations in those subjects receiving the active study material [17]. It is highly possible that more cases of empyema will be seen in patients on treatment with similar regimens.

Standard treatment of empyema involves appropriate antibiotic therapy as well as tube drainage initially. There is controversy, however, regarding the use of fibrinolytic agents in the treatment of empyema. A large, multicenter trial in adults did not demonstrate an advantage to adding streptokinase to the pleural space with regards to outcome, rate of surgery, or length of hospital stay [18]. In earlier studies the use of these agents did appear to confer a lower risk for surgical intervention. A recent Cochrane Review did not find any mortality benefit from intrapleural fibrinolytics [19]. However, this patient responded very well to instillation of tPA through her pleural catheter. We believe this obviated the need for more aggressive surgical intervention such as Video-Assisted Thoracoscopic Surgery (VATS). A blinded, 2-by-2 factorial trial in 210 adult patients found that intrapleural tPA-DNase treatment improved fluid drainage in patients with pleural infection and reduced the frequency of surgical referral and the duration of hospital stay [20]. The success of intrapleural fibrinolysis in this patient may be due to its administration early in the course of her empyema, when the fluid has formed loculations (fibrinopurulent phase) but has not organized yet. Although fibrinolytics may themselves cause weeping of the pleural surface and thus an increase in chest tube drainage, this patient's rapid recovery following instillation of tPA implies a therapeutic role for this agent at the fibrinopurulent phase. If this patient had a lack of response to the tPA or well-organized empyema, we would have proceeded with a thoracotomy with formal decortication.

This case illustrates the importance of recognizing empyema as a potential complication in CF. Physicians should have a heightened index of suspicion for parapneumonic disease in CF patients receiving immunosuppressant agents, even at low dose. This is of increasing concern as more anti-inflammatory agents such as statins, peroxisome proliferator-activated receptor gamma agonists, and neutrophil antagonists enter the CF therapeutic pipeline [16].
